# A novel approach for the identification of cadmium-chelating compounds in plant-based foods using SEC-ICP-MS/MS and SEC-QTOF-MS

**DOI:** 10.1007/s00216-025-05931-y

**Published:** 2025-06-05

**Authors:** Julian Cardini, Jens J. Sloth, Katrin Loeschner

**Affiliations:** Research Group for Analytical Food Chemistry, National Food Institute, Technical University of Denmark, Kgs. Lyngby, DK‑2800 Denmark

**Keywords:** Multielement speciation, SEC-ICP-MS/MS, SEC-QTOF-MS, Cadmium, Plant-based foods

## Abstract

The increasing trend towards more plant-based diets has underscored the need for a comprehensive understanding of the presence of potentially toxic elements in plant-based foods. Among these, cadmium is of particular concern due to its potential health risks. Our understanding of how cadmium is bound in plant-based foods and how this affects its bioavailability is currently limited. This paper introduces a qualitative methodology that combines SEC-ICP-MS/MS and SEC-QTOF-MS for identifying cadmium-chelating compounds and performing simultaneous speciation analysis of phosphorus, sulfur, calcium, iron, and zinc in plant-based foods. The present study details the optimization of the cadmium extraction process across seven different matrices with varying protein content. A large variation in the recoveries, ranging from 2.3 to 72.3%, was observed, suggesting strong cadmium binding to certain foods. The study employed two size exclusion columns (optimal ranges 0.1–7 kDa and 10–600 kDa), which were optimized for chromatographic peak resolution and analysis time to separate the extracted compounds. The study identified distinct cadmium-chelating patterns in plant-based foods, especially within the 1–20 kDa range. The multielement speciation data were used to detect proteins and other compounds, including phytic acid, phytochelatins, and metabolites, highlighting their chelating properties. The methodology established in this paper represents a significant step forward in cadmium speciation in plant-based foods and holds promise for future bioaccessibility studies.

**Supplementary Information:**

The online version contains supplementary material available at 10.1007/s00216-025-05931-y.

## Introduction

Cadmium (Cd) is a non-essential, potentially toxic element that has become a growing concern due to its widespread environmental contamination. Industrial activities, including mining, smelting, manufacturing, and combustion of fuels, have significantly contributed to the release of Cd into the environment. Additionally, the use of phosphate fertilizers and the application of treated sewage sludge, known as biosolids, in agriculture further amplify Cd contamination.

Consequently, Cd has infiltrated agricultural ecosystems, resulting in its uptake and accumulation in various crops, including those consumed as part of a plant-based diet [1–3]. Plant-based foods, which are typically perceived as healthy, may inadvertently expose individuals to Cd. Leafy greens, such as spinach [4] and lettuce [5], and cereal crops, including rice [6] and wheat [7], are some of the main contributors to dietary Cd exposure [8]. Exposure to Cd through contaminated food sources can lead to a range of detrimental health effects. Long-term ingestion of Cd has been linked to renal dysfunction, skeletal abnormalities, reproductive disorders, and carcinogenicity [1].

As plant-based foods are a major source of nutrients for many populations, understanding the sources, transport, and accumulation of Cd in these foods is crucial for developing effective strategies to mitigate the health risks associated with Cd exposure. There are significant knowledge gaps in our understanding of the speciation of Cd in plant-based foods. Speciation refers to the different forms in which an element exists, such as its chemical form or its binding to other molecules. These forms can have different toxicities and bioavailabilities. Several compounds within plant-based foods have shown the ability to bind Cd and hence reduce its bioavailability, including phytochelatins (PCs), metallothioneins (MTs), organic acids (e.g., citric acid) [9], and dietary fibers [10]. PCs are small, cysteine-rich peptides that play a crucial role in the detoxification of heavy metals in plants [11].

PCs have a high affinity for binding Cd and other heavy metals, forming stable chelates that sequester these toxic ions and prevent them from interfering with essential cellular processes. PCs consist of three amino acids: glutamine (Glu), cysteine (Cys), and glycine (Gly) [12–14]. PCs form a family of structures with 2 to 5, but can be as high as 11, repetitions of the -Glu-Cys dipeptide units. Based on the number of -Glu-Cys units, PCs have been classified as PC2, PC3, PC4, PC5, and PC6, etc. [12, 15]. PCs have been identified in a wide variety of plant species and in some microorganisms. MTs are low molecular weight, cysteine-rich proteins, weighing approximately 6500 Da, with their weight varying based on the number of bound metals. These proteins bind metal ions through thiolate clusters, enabling them to play a crucial role in detoxification. By sequestering toxic metal ions such as Cd, MTs prevent cellular damage and protect plant cells from toxicity. Additionally, their antioxidant properties help mitigate oxidative stress.

While low molecular weight (LMW, < 10 kDa) chelators like PCs have been studied to some extent, high molecular weight (HMW, > 10 kDa) Cd-binding proteins remain insufficiently characterized in plants. Emerging evidence suggests that proteins, ranging from 40 to 80 kDa [16–18], contribute significantly to Cd detoxification in plants. However, previous methodologies have lacked the resolution and sensitivity to analyze complexes with a higher molecular weight.

Size exclusion chromatography (SEC) coupled with inductively coupled plasma-mass spectrometry (ICP-MS) has previously been used to study Cd species in plants [13, 17–22]. All studies used SEC columns with an optimal separation range between 100 and 7000 Da and primarily focused on PCs and not larger proteins. These methods lacked the capability to detect and resolve Cd-binding proteins above 70 kDa. SEC offers the advantage of separating species based on their size. However, its resolution and separation range are limited, potentially leading to less precise identification of individual compounds when relying on molecular weight information (obtained via column calibration) only. Therefore, molecular mass spectrometry (MS) was typically applied for identification of the species. Direct infusion electrospray ionization MS (DI-ESI-MS) of collected SEC fractions was used in some of the studies [16, 21]. These fractions had typically a broad molecular weight range, which might have limited the ability to distinguish different peaks [19, 23]. Most studies analyzed PCs in collected SEC fractions using reversed phase chromatography [16–18], capillary zone electrophoresis [13], and ion pair chromatography [20] hyphenated to MS detection methods.

Essential trace elements such as iron (Fe), zinc (Zn), and calcium (Ca), especially deficiencies of those, have been shown to affect the bioavailability of Cd. It is therefore of interest to study not only the speciation of Cd in plant-based foods but also of these elements [24]. Persson et al. [19] applied multi-elemental speciation analysis using SEC-ICP-MS and ESI-TOF-MS to study barley genotypes with different tolerance to Cd toxicity. They found that the PCs induced by Cd toxicity also bound several essential trace elements, including Zn, copper (Cu), and nickel (Ni), whereas no manganese (Mn) species were detected. They observed “highly variable affinities” of the elements and major differences between Cd and Zn despite being closely related elements. These findings highlight that multi-element speciation is not just analytically valuable but biologically and nutritionally meaningful. By studying which elements share binding partners, we can better understand competitive interactions between toxic and essential metals, assess potential nutrient displacement, and characterize metal-specific detoxification mechanisms. This level of resolution is critical for both food safety evaluations and the development of Cd-resilient crop varieties through informed breeding.

The methodology presented in this study integrates SEC-ICP-MS/MS and quadrupole time-of-flight mass spectrometry (QTOF-MS) for a comprehensive qualitative analysis of Cd-chelating compounds. Furthermore, this study investigates Zn, Fe, and Ca alongside Cd to assess in how far they compete for the same chelating compounds. Unlike previous SEC-ICP-MS studies, this method extends the separation range by employing two SEC columns with distinct optimal separation ranges, spanning from 600 to 10 kDa and from 10 kDa to 100 Da. This broad molecular weight range enhances resolution and allows for the detection of both LMW and HMW Cd-binding species. QTOF-MS offers exact mass-to-charge ratio measurements, enabling the identification of proteins through accurate mass determination and selective fragmentation.

## Materials and methods

### Chemicals

The eluents for liquid chromatography (LC) were composed of purified water, acetonitrile, and methanol. Ultrapure water (UPW) was sourced from a Milli-Q Advantage water purification system (Merck Millipore, Burlington, MA, USA). Both acetonitrile and methanol, of LC-MS grade, were supplied by Honeywell Riedel-de Haën (Seelze, Lower Saxony, Germany). Concentrated nitric acid (67–70%) for the digestion of samples, of PlasmaPure grade, was bought from SCP Science (Baie-d’Urfé, Quebec, Canada). Ammonium acetate and formic acid, used for sample extraction, were also of LC-MS grade and bought from Merck (Supelco, Bellefonte, PA, USA). Reagent grade ethylenediaminetetraacetic acid (EDTA) was also purchased from Merck. For calibrating the Superdex 200 Increase chromatography, a protein standard mix with molecular weights ranging from 15 to 600 kDa was obtained from Sigma-Aldrich (Supelco, Bellefonte, PA, USA). Additionally, the calibration of the Superdex 30 Increase utilized four standards: horse heart myoglobin (CAS No. 100684-32-0), bovine insulin (CAS No. 11070-73-8), [Glu1]-Fibrinopeptide B (CAS No. 103213-49-6), and raffinose (CAS No. 17629-30-0), all included in the proteomics kit from Waters (Milford, MA, USA). The elemental standards for calibration and optimization of the ICP-MS, consisting of Cd, Rh, Zn, Ca, Fe, P, and S at a concentration of 1000 mg/L, were procured from SCP Science (PlasmaCAL, Baie D’Urf’e, Quebec, Canada).

### Samples

The methodology was developed using samples that were purchased in powdered form apart from the black-eyed beans and beluga lentils. Both samples were homogenized using a Retsch GM 200 (Haan, North Rhine-Westphalia, Germany) at 10,000 RPM for 1 min, followed by sieving through a Retsch AS200 with a 500 μm mesh. Samples were acquired from Danish internet stores.

The samples were then classified into three categories based on the protein content in their hydrated state (Table S1 in Online Resource 1): (a) high protein content (>5 g/100 g): This category included black-eyed beans and beluga lentils; (b) medium protein content (2–5 g/100 g): Samples in this group consisted of tigernut and basmati rice; and (c) low protein content (<2 g/100 g): This group comprised sweet potato and beetroot leaves. The nutritional values from the nutrition declaration are presented in Table S2 in Online Resource 1.

### Acid digestion and total Cd measurement with ICP-MS/MS

Approximately 0.3 g of each sample was weighed into 18-mL quartz tubes, and 1 mL of ultrapure water and 4 mL of concentrated HNO_3_ (67–69%) were added to the tubes. The samples were then subjected to a digestion program that involved ramping up to 250 °C over a period of 10 min, maintaining this temperature for 20 min, and then allowing the samples to cool down for 30 min using a Multiwave 7000 microwave oven (Anton Paar GmbH, Graz, Austria). After digestion, the digested samples were transferred into 50-mL disposable polypropylene tubes, and ultrapure water was added to bring the total volume up to 25 mL. The samples were then diluted 10 times in 2% HNO_3_ in preparation for analysis. To ensure the quality of the analysis, procedural blank samples as well as certified reference materials, SRM-1572 citrus leaves (National Institute of Standards and Technology, NIST, Gaithersburg, USA) and DORM5 Fish Protein (National Research Council Canada, NRC, Ottawa, Canada), were included in the analysis. Samples were prepared in triplicates. Determination of Cd mass concentrations was performed based on external calibration by measuring the Cd standards in the concentration range from 0 to 25 μg/L with internal standardization (1 μg/L Rh). Calibration and internal standards were prepared from standard solutions that contained 1000 mg/L Cd or Rh (SCP Science, PlasmaCAL, Baie D’Urf’e, Quebec, Canada). Measurements were performed on the Agilent 8900 ICP-MS/MS instrument using an SPS 4 autosampler in single quadrupole mode measuring ^114^Cd using no cell gas.

### Extraction optimization

The extraction procedure was adapted and modified from the methodology presented by Persson et al. for the multielement speciation of barley genotypes [19]. One hundred mg of dry sample were weighed into a 2 mL Eppendorf tube. Subsequently, 2 mL of UPW with 50 mM ammonium acetate was added to the sample. The samples were then subjected to vigorous vortexing to ensure complete wetting, followed by ultrasonication using a Branson 5800 ultrasonic bath (Fisher Scientific Biotech Line, Roskilde, Denmark) for a duration of 30–60 min. Post-ultrasonication, the samples were centrifuged at a force of 10000 g for a period of 15 min using an Ole Dich microcentrifuge type 157 MP (Ole Dich Instrumentmakers. ApS, Hvidovre, Denmark). The supernatant obtained was removed and ready for measurement. During the optimization of the extraction process, several parameters were tested. These included the extraction time, with durations of 30, 45, and 60 min being evaluated, and the content of acetonitrile and methanol in the extraction solvent, which was varied between 10%, 20%, and 30% with an extraction time of 60 min. A full factorial design of experiment (DOE) approach was not feasible due to the complexity: six different samples and four parameters, with three levels in triplicates would have to be measured, resulting in 1458 samples analyses. Consequently, each parameter was evaluated individually, hindering the ability to examine their interdependent effects.

### Chromatographic separation and optimization

The extracted compounds were chromatographically separated using two distinct columns, namely the Superdex 30 Increase (3.2 × 300) and the Superdex 200 Increase (3.2 × 300) by Cytiva (Marlborough, MA, USA). The liquid chromatography (LC) system employed for this process was composed of an Agilent 1290 binary pump, a 1290 autosampler with a thermostat, a 1290 column oven, and a 1260 MWD detector that measures at 280 nm, hyphenated to a Bruker Maxis 4G QTOF. A second LC system was also utilized and consisted of an Agilent 1260 binary pump and a 1260 autosampler with both an integrated thermostat and column oven. This system was hyphenated to an Agilent 8900 ICP-MS/MS with a MicroMist nebulizer, a Scott-double-pass spray chamber cooled to 2 °C, a 2.5 mm torch, and a nickel skimmer cone. The mobile phase utilized for both columns consisted of 50 mM ammonium acetate dissolved in UPW. During the use of the QTOF-MS, the mobile phase was supplemented with 0.1% formic acid. During the optimization phase of the chromatographic process, flow rates ranging from 0.050 to 0.10 mL/min were tested in 0.025 mL/min increases to achieve the optimal separation of compounds. Injection volume was set to 10 µL on both systems.

### ICP-MS/MS and QTOF-MS parameters

Table 1 shows the instrument parameters for both the Agilent 8900 ICP-MS/MS and the Bruker Maxis 4 g QTOF.

**Table 1:** ICP-MS/MS and QTOF-MS parameters

ICP-MS/MS		QTOF-MS	
RF power (W)	1550	End plate offset voltage (V)	500
Sampling depth (mm)	8	Capillary voltage (kV)	4.5
Nebulizer gas flow (L min^−1^)	1.1	Nebulizer gas pressure (bar)	0.4
Spray chamber temperature (°C)	2	Dry gas flow rate (l/min)	4
O_2_ flow rate (mL/min)	0.35	Drying gas temperature (°C)	180
Octopole bias voltage (V)	−3	Scan mode	Positive
Axial acceleration voltage (V)	1.5	Mass range (m/z)	300–3700
Cell exit voltage (V)	−60	Spectra rate (Hz)	0.2

In order to optimize the signal of the elements during an LC-ICP-MS/MS run, initially the lenses and nebulizer gas flow were tuned using a standard solution as recommended by the manufacturer. Then the mobile phase, which consisted of 50 mM ammonium acetate in UPW, was spiked with specific concentrations of various elements (100 ng/mL of sulfur (S), phosphorus (P), and calcium (Ca), and 10 ng/mL of Cd, iron (Fe), and zinc (Zn)) and injected into the system. To minimize interferences as well as enhance the signal, different amounts of oxygen were introduced into the collision/reaction cell, and the signals were measured. Ca, Zn, and Cd were measured “on mass” and P, S, and Fe were measured by “mass shift” approach as recommended in the literature [22, 25]. The selected isotopes are presented in Table 2. For a direct comparison of the samples measured, a post-column infusion of rhodium (Rh) as an internal standard at a concentration of 50 µg/L was performed.

**Table 2:** First quadrupole (Q1) and second quadrupole (Q2) mass-to-charge ratios (m/z) for the selected elements during the LC-ICP-MS/MS analysis

Element	m/z Q1	m/z Q2
P	31	47
S	32	48
Ca	44	44
Fe	56	72
Zn	66	66
Cd	114	114

### Repeatability of the method

Repeatability of the method was estimated by analysis of three different samples with three different protein contents: black-eyed beans, tigernuts, and beetroot leaves. Factors that were assessed included intra/inter-day repeatability of the extraction and intra-day method repeatability in terms of retention times and peak areas for all detectors. Extraction repeatability tests were performed on triplicate extracts on two subsequent days. The extracts were diluted to a dilution factor of 200 with 2% HNO_3_. The elements tested for were S, P, Zn, Fe, Ca, and Cd, where the ratio of signals from the analyte and internal standard was used to determine repeatability. The SEC-ICP-MS/MS and SEC-QTOF-MS method repeatability was estimated using both Superdex 30 Increase and Superdex 200 Increase columns using duplicate measurements. The repeatability of various factors, including the retention time (RT) and peak area (for UV, ICP-MS/MS, and QTOF-MS detection), of the sample peaks was assessed.

### Data analysis

Data analysis was performed using Agilent Mass Hunter 5.1 (Santa Clara, CA, USA) for the ICP-MS/MS measurements and for QTOF-MS as well as for UV measurements Bruker Compass DataAnalysis 4.4 (Billerica, MA, USA). Peak integration on both softwares was performed manually. Statistical analysis was performed in Microsoft 365 Excel version 2408 (Redmond, WA, USA) and figures were created using OriginPro 2023 (OriginLab Corporation, Northampton, MA, USA). Molecular structures were created using ChemDraw The graphical abstract was created using BioRender (Toronto, Ontario, Canada [26]).

## Results and discussion

### Optimization of extraction

**Fig. 1:**
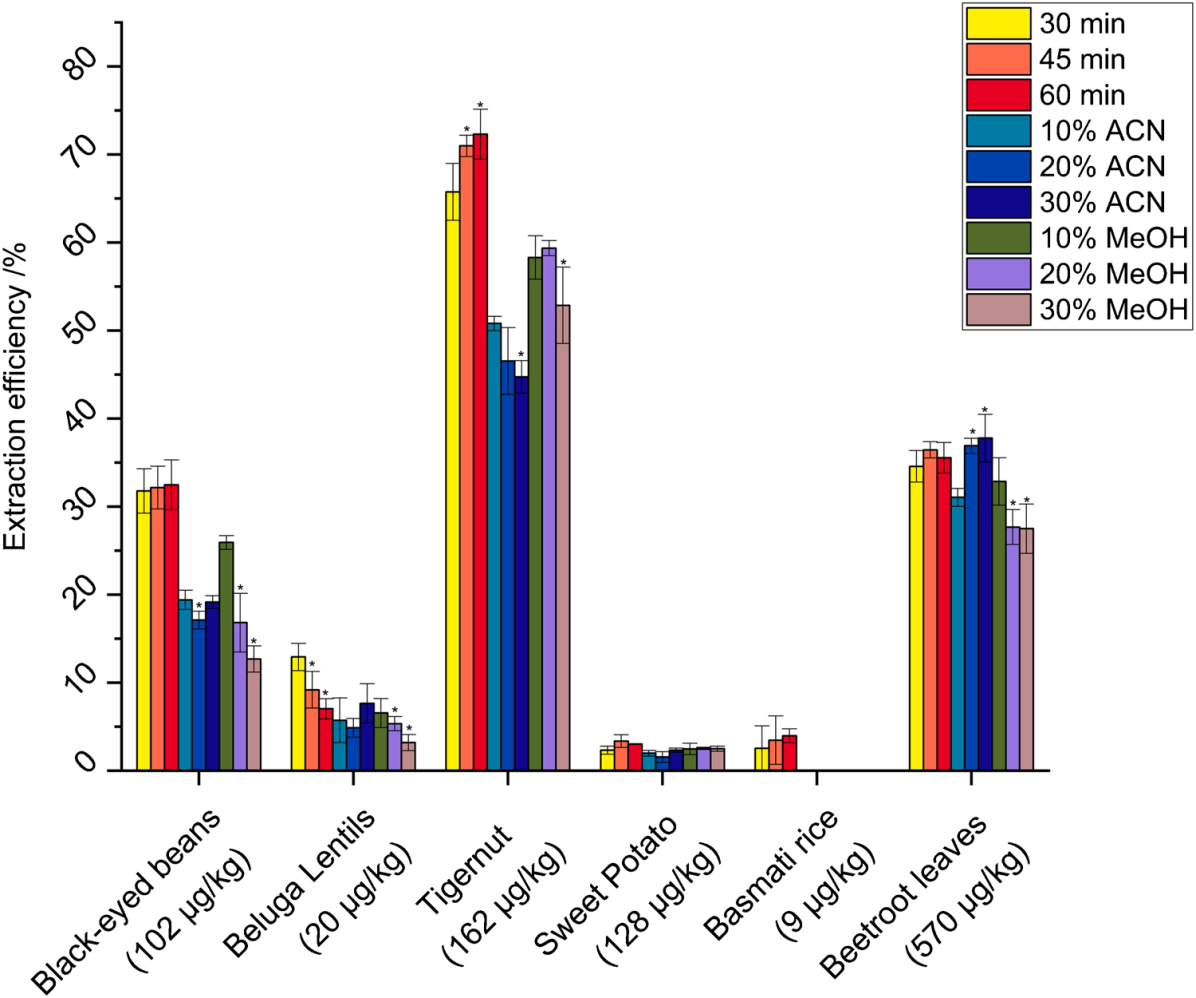
Comparative analysis of Cd extraction efficiency percentages in different plant-based foods under various extraction conditions. The graph illustrates the efficiency of Cd extraction across different time intervals (30–60 min, 50 mM ammonium acetate only) and solvent compositions (60 min extraction time, 50 mM ammonium acetate with addition of varying amounts of acetonitrile/ACN or methanol/MeOH). Measurements were taken in triplicate. The error bars represent one standard deviation. The value in parentheses represents the mass concentration of Cd in the samples. The asterisk shows a significant change in comparison to the first extraction focusing on this parameter (*t*-test, *p* < 0.05)

The aim of the extraction was to recover the broadest range of Cd species possible while preserving their native state, referring to their natural, functional conformation. Figure 1 shows the extraction efficiency percentage of naturally occurring Cd for all six samples in comparison to a microwave-digested sample. Total Cd mass fractions of the samples were in the range of 9 to 570 µg/kg with the lowest concentrations in basmati rice and the highest concentrations in beetroot leaves. Extraction recoveries ranged from 1.6% for sweet potato to 72.3% for tigernuts, with relative standard deviations ranging from 0.04 to 4.3% based on triplicate analysis. It is evident from the graph that the recovery percentages varied significantly across the samples. For instance, tigernuts, which have a relatively high fat content, showed a higher recovery percentage compared to other samples such as sweet potato, which had the highest starch content, together with rice, which had the lowest recovery. This could be attributed to the inherent differences in the composition and structure of these matrices, which influence the extractability of Cd. In addition, the production process of the samples could also have an influence since sweet potatoes were cooked, dried, and ground to a flour, and nuts were only ground.

Recovery percentages for black-eyed beans, tigernuts, and basmati rice showed a general trend of increasing with extraction time (30–60 min), which aligned with the expectation that a longer extraction time allows for more thorough penetration of the solvent into the matrix. However, this trend was not as pronounced in other samples such as sweet potato, beetroot leaves, and beluga lentils, even decreasing for the latter. The addition of both acetonitrile (10–30%) and methanol (10–30%) was tested with the aim to disrupt the plant cell walls and release the Cd stored in the vacuoles. However, the results suggest that the use of organic solvents in general decreased the recovery of Cd. The diminished recovery could be attributed to the fact that Cd is bound to proteins and peptides, which have generally a lower solubility in organic solvents compared to water [27]. Due to the low recovery in three samples (beluga lentils, sweet potato, and basmati rice), the volume of the solvent was increased to 10 mL to increase the solvent to sample ratio, but the extraction recovery of cadmium did not increase. Furthermore, probe sonication with the same solvent to sample ratio was tested with the same results (data not presented).

The extraction process, utilizing 50 mM of ammonium acetate with a duration of 30 min under ultrasonication, was established as sufficient. In general, protein extraction recoveries range from 30 to 70%, but are highly matrix dependent [28]. While extended extraction times did result in a better recovery for some of the samples, the difference was not substantial enough to warrant a longer extraction period. Persson et al. [19] found similar cadmium recoveries ranging from 20% from barley roots to 60% from barley shoots. In the literature, it is commonly acknowledged that buffer extractions are efficient for protein chelates [13, 16–19, 23]. A water extraction including buffer with concentrations between 10 and 100 mM is commonly used, since the Cd chelates are stable around a neutral pH [29]. While alternative buffers such as tris(hydroxymethyl)aminomethane hydrochloride (Tris-HCl) or 4-(2-Hydroxyethyl)piperazine-1-ethane-sulfonic acid (HEPES) could potentially improve extraction efficiency and enhance separation in SEC, they are not compatible with our analysis due to their non-volatility. Their non-volatile nature means they do not efficiently evaporate during the ionization process, which can result in adduct formation, unstable spray conditions, and contamination of the MS source, requiring frequent maintenance [30]. For ICP-MS, Tris-HCl has been shown to block the plasma torch after repeated runs and to block the nebulizer twice as fast as ammonium nitrate, leading to unwanted additional maintenance of the ICP-MS instrument [31]. As this work aims at analyzing a larger number of samples and uses long analysis times, this would be a challenge. According to Lago et al. [31], ammonium sulfate, HEPES, and MOPS mobile phase buffers should not be used in the separation and subsequent quantification of metalloproteins through SEC-ICP-MS, as they alter metal species profiles.

### Optimization of chromatographic conditions

The choice of the Cytiva Superdex 30 and Superdex 200 columns was based on the specific requirements of the analysis. These columns had different optimal separation ranges, making them suitable for a wide mass range of analytes. The Superdex 30 Increase column had an optimal separation range of 100 to 7000 Da and was adequate for peptides and small proteins. On the other hand, the Superdex 200 Increase column, with its optimal separation range of 10 to 600 kDa, was suited for larger proteins. As mentioned previously, the mobile phase for the separation of these compounds was 50 mM of ammonium acetate in UPW. The addition of 0.1% of formic acid to the mobile phase when the samples were measured with the QTOF-MS was necessary, as it was observed to enhance ionization [32]. Moreover, the addition of formic acid did not result in shifts in retention time.

The results of the flow rate optimization demonstrated that lower flow rates generally improved resolution, consistent with the principles of size exclusion chromatography. For example, the resolution for horse heart myoglobin improved from 1.46 at a flow rate of 0.1 mL/min to 1.73 at 0.05 mL/min. Similarly, for bovine insulin, the resolution improved from 1.18 at 0.1 mL/min to 1.45 at 0.05 mL/min. This trend was also observed for Glufib, with the resolution improving from 1.91 at 0.1 mL/min to 2.05 at 0.05 mL/min. Table S3 in Online Resource 1 presents the full width at half maximum (FWHM) and resolution for different analytes (horse heart myoglobin 16,952 Da, bovine insulin 5733 Da, Glufib 1570 Da, raffinose 527 Da) under three different flow rates (0.1 mL/min, 0.075 mL/min, 0.05 mL/min).

Similar to the Superdex 30 Increase, the results of the optimization of the Superdex 200 Increase column showed a trend whereby lower flow rates generally improved resolution. However, for the Superdex 200 Increase column, all analytes showed increased FWHM with decreasing flow rates, indicating broader peaks. For example, thyroglobulin bovine’s resolution slightly decreased from 2.04 to 1.97 when the flow rate was reduced from 0.075 to 0.05 mL/min, due to the increase in FWHM from 1.40 to 2.21 min. This suggests that the peaks had broadened to an extent that negatively impacted the resolution due to longitudinal diffusion. Table S4 in Online Resource 1 shows the FWHM and resolution for different analytes (thyroglobulin bovine 670 kDa, γ-globulins 150 kDa, ovalbumin 44.3 kDa, ribonuclease A type I-A 13.7 kDa, and p-aminobenzoic acid 137 Da) under three different flow rates (0.1 mL/min, 0.075 mL/min, 0.05 mL/min).

For both columns, the optimal flow rate was determined to be 0.075 mL/min, providing a good compromise between resolution and analysis time. This flow rate represents a 20% increase in resolution compared to the lowest flow rates for both columns, allowing for efficient separation of analytes while keeping the analysis time to 60 min per sample.

### Optimization of ICP-MS/MS

ICP-MS/MS in mass shift mode with oxygen as reaction gas allows detection of P and S (as ^31^P^16^O^+^ and ^32^S^16^O^+^) with enhanced sensitivity and selectivity [25]. The influence of the oxygen gas flow in the reaction cell on all elements of interest was investigated. Figures S1 and S2 in the Online resource 1 present the signal-to-noise ratio (S/N) normalized by concentration for P, S, Ca, Fe, Zn, and Cd, with oxygen flow rates ranging from 20 to 80%. The signal of P and S showed an increase up to flow rates of 0.25 mL/min and 0.35 mL/min, respectively, before declining. This trend suggests that the formation of ^31^P^16^O+ and ^32^S^16^O+ ions is optimal at these flow rates, enhancing sensitivity and selectivity, and agrees with previous publications using similar instrumentation [25]. Ca exhibited a consistent decrease in signal intensity after 0.25 mL/min. Fe, Zn, and Cd displayed a similar pattern to S, with a maximum intensity at 35% followed by a constant decrease.

One instrumental setting has to be chosen for time-resolved analysis, and it is necessary to select a compromise condition that will provide the most reliable and consistent results across all elements. Based on the measured data, an oxygen flow rate of 35% appeared to be the most suitable choice. At this flow rate, the highest possible peak intensities for S, Fe, Zn, and Cd were observed, suggesting optimal ion formation and enhanced sensitivity and selectivity for these elements. While Ca and P do not reach their optimum peak intensities at this flow rate, the signal intensity was still reasonably high, ensuring adequate detection. SEC-ICP-MS/MS chromatograms of blank in comparison to a selected sample (black-eyed beans) are presented in Fig. S3 in the Online resource 1 to demonstrate the efficient removal of the background signal for all selected elements.

### Repeatability of the extraction and the SEC-ICP-MS/MS and SEC-QTOF-MS method

The RSD values for intra-day repeatability of the extraction for all elements (P, S, Ca, Fe, Zn, Cd) across all samples were between 0.1 and 10.8%, with an average of 2.1% based on the analysis of triplicates (*N*=3) (see also Table S7 in Online Resource 1). The RSD values for inter-day repeatability (based on analysis on 2 days) showed some variability, but the average RSD values for all elements and samples remained below 13%, indicating satisfactory inter-day repeatability of the extraction.

The performance of the Superdex 30 Increase column was shown to be consistent, with RSD for retention time across all detectors and samples ranging from 0.0 to 0.9% (see Table S5 in Online Resource 1). UV detection demonstrated good repeatability across all samples. The average RSD for peak area was 5.3% for black-eyed beans, 2.5% for tigernuts, and 5.1% for beetroot leaves. Largest variations (RSD up to 14.8%) were observed for beetroot leaves. The QTOF-MS detection also showed good repeatability across all samples. The average RSD for peak area was 5.9% for black-eyed beans, 3.0% for tigernuts, and 5.1% for beetroot leaves. Highest variation was observed for tigernuts (up to 16.5%) using ICP-MS/MS detection, whereas ICP-MS/MS in general presented the lowest average RSD values across all samples and detection methods. The average RSD for peak area was 4.9% for black-eyed beans, 2.9% for tigernuts, and 2.4% for beetroot leaves. Using ICP-MS/MS, the largest number of peaks was found due to two main reasons: All species of the six elements were counted as different peaks, and ICP-MS/MS has the highest sensitivity and selectivity among the detectors.

Also, the performance of the Superdex 200 Increase was shown to be consistent, with RSD values for retention time across all samples ranging from 0.0 to 0.4% (see Table S6 in Online Resource 1). The UV detection method demonstrated good repeatability across all samples. The RSD was 5.3% for black-eyed beans, 2.5% for tigernuts, and 3.3% for beetroot leaves. However, the variability in the UV measurements suggests that factors such as detector response or sample preparation inconsistencies might be influencing the results. The ICP-MS/MS detection method demonstrated excellent repeatability across all samples. The average RSD was 4.9% for black-eyed beans, 2.9% for tigernuts, and 2.4% for beetroot leaves.

It has been reported in the literature that achieving reproducibility for the speciation of Cd chelates is challenging due to the excessive retention of free metal ions on the column, which can lead to ligand exchange and destabilization of the Cd-complexes [19]. To address this issue, 10 µL containing 5 mM EDTA was injected three consecutive times after the analysis of the black-eyed bean sample on the Superdex 30 Increase column. It could be demonstrated that only a small amount of Cd was retained on the column (Fig. S4. in Online Resource 1). The bound metal did not interfere with repeatability, likely due to two main factors: First, the samples separated on the SEC columns generally contained 10,000 times lower Cd concentrations than the ones studied in the literature [19]. Second, the columns used in previous studies were older versions of the same column, which likely had a higher affinity for these metals. Nevertheless, it is advisable to inject EDTA to clean the column of adsorbed metals after each analytical run.

### Application of the final method

There was in general a good agreement between the molecular masses determined by column calibration and QTOF-MS (by measuring the exact m/z) for molecular weights up to 25 kDa. Consequently, the need for a column calibration was eliminated for the Superdex 30 Increase column.

**Fig. 2:**
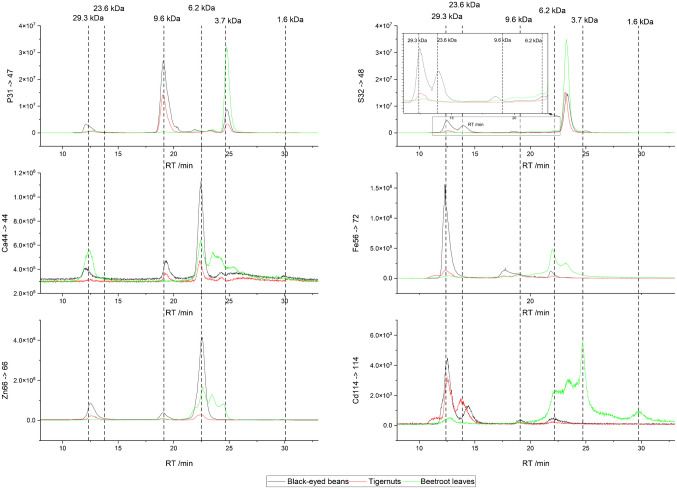
SEC separation and ICP-MS/MS detection of P, S, Ca, Fe, Zn, and Cd in cps (in one analytical run) using Superdex 30 Increase of the three samples black-eyed beans, tigernuts, and beetroot leaves. Vertical lines represent the peaks identified in the chromatograms for the Cd signal and the corresponding molecular weights (based on size calibration of the column)

A sample set containing black-eyed beans, tigernuts, and beetroot leaves samples was analyzed using both SEC-ICP-MS/MS and SEC-QTOF-MS. The three samples chosen were based on the categories: one from high protein, one from medium protein, and one from low protein samples to demonstrate the method’s capability and applicability on a broad variety of plant-based samples. Figure 2 presents the chromatograms of these samples, which were obtained using the Superdex 30 Increase coupled to ICP-MS/MS. Using a post-column infusion of internal standard with Agilent's point-to-point correction, a comparison of the relative distribution of the elements between the samples can be made. Both, P and S, can be used as markers for proteins as Cd binds to thiol groups of the amino acid cysteine, and often proteins can be phosphorylated post-translationally [33].

The first peak in the Cd signal, eluting at 12.3 min, appeared for all other element traces and had a mass of approximately 29.3 kDa, based on SEC column calibration. This peak likely corresponds to proteins that surpass the efficient separation size limit (0.1–7 kDa) of the Superdex 30 Increase. In agreement with that, a pronounced peak can be found in the S signal. Notably, Cd and Fe seemed to bind predominantly to this fraction in the black-eyed beans and tigernuts sample.

The second peak in the Cd signal (13.7 min), especially pronounced for the tigernuts samples and, with a slight delay, in the black-eyed beans sample, showed a corresponding peak in the S signal. No corresponding peak was observed for Ca, Fe, or Zn. It had a molecular weight of 25.6 kDa as measured with QTOF-MS and 23.6 kDa as determined by the SEC column calibration. Literature suggests that such proteins could be MTs. Xie et al. [34] and Mesna et al. [35] have reported Cd-chelating MTs with molecular weights of 23 to 27 kDa in both fish and humans, but not in plants. Generally, plant MTs are produced with a molecular weight range of 4–8 kDa and the observed MTs could therefore be dimers or trimers [36–38].

**Fig. 3:**
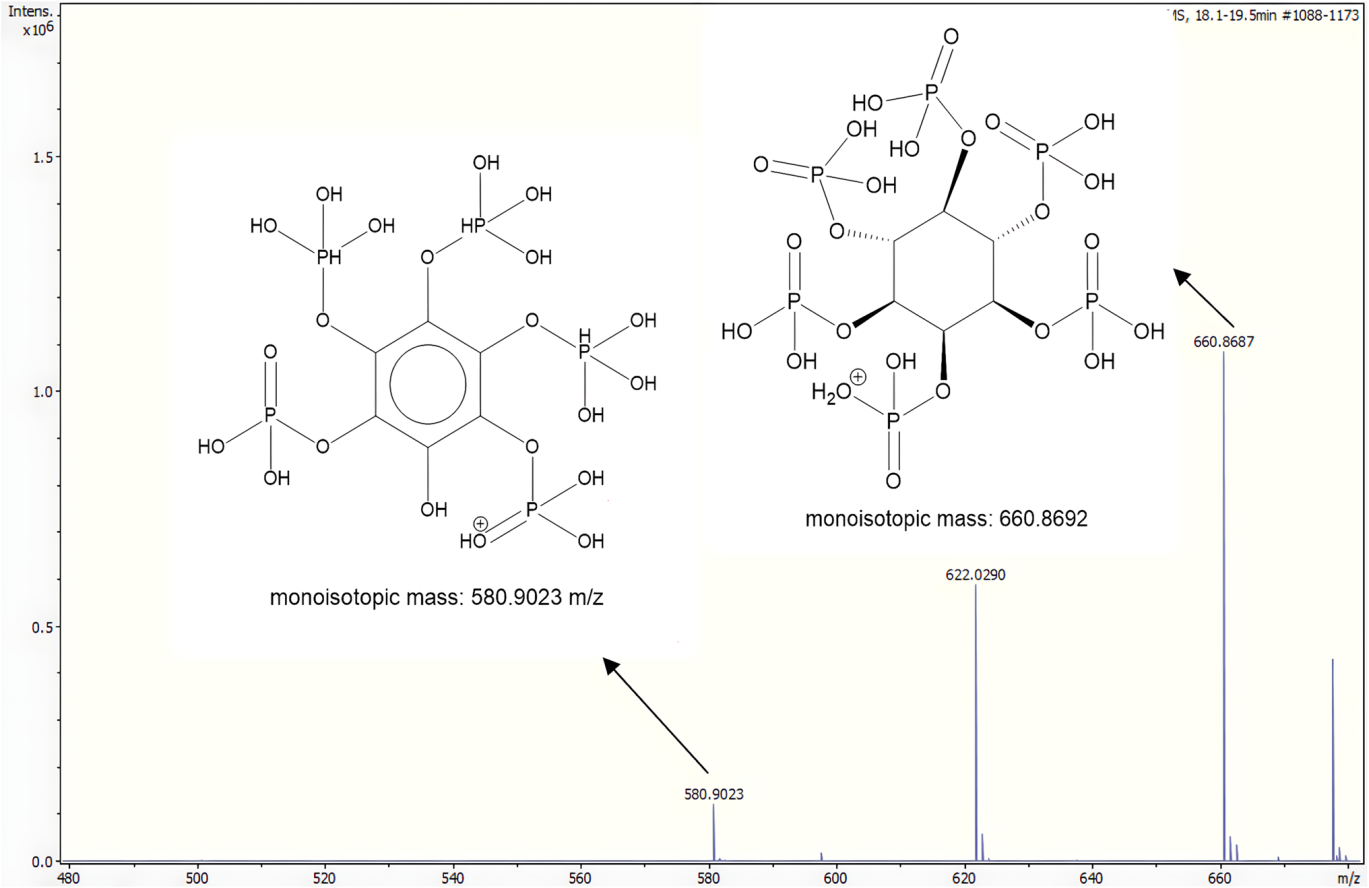
Summed mass spectrum obtained from QTOF-MS in positive mode between 18.1 and 19.5 min. The mass spectrum ranges from 480 to 680 m/z, highlighting the monoisotopic mass of phytic acid at 660.8687 m/z and a fragment ion at 580.9023 m/z. Fragments and structures were predicted in silico by CMF-ID [39]

The third peak in the Cd signal (19.3 min) was prominent in the P trace as well as in the Zn and Ca traces of the black-eyed beans and tigernut samples. Additionally, Fe exhibited a broad peak. The molecular weight of this peak was determined to be 9.6 kDa through column calibration. Given the low S signal, it can be inferred that this peak is neither a peptide nor a protein, but rather a compound with a high P content and the ability to bind various bivalent metals. The significant P peak suggests the presence of multiple phosphorus groups, such as those found in phytic acid. The mass spectrum acquired with QTOF-MS at the same retention time (Fig. 3) displays the [M+H]^+^ peak of phytic acid at 660.8687 m/z with 0.8 ppm deviation from theoretical monoisotopic mass. Typically, this compound is analyzed in negative ESI mode due to its numerous phosphate groups, but since proteins and peptides get ionized better in positive mode, it was acquired in positive ESI mode. To confirm its identity as phytic acid, in silico fragmentation was performed using CMF-ID 4.0 [39], identifying three fragments, one of which is visible in Fig. 3 at 580.9023 m/z (0.03 ppm deviation). Two further fragment peaks at m/z 562.8917 (1.6 ppm deviation) and 500.9359 (0.04 ppm deviation) were present. Based on this data, the compound was identified at level 3 on the identification scale proposed by Schymanski et al. [40]. To achieve a level 1 identification, a measurement in negative mode and a phytic acid standard should additionally be used to match the retention time, monoisotopic mass, and fragmentation pattern during the MS/MS experiment.

The fourth peak in the Cd signal (22.1 min) appeared also clearly in the Fe, Zn, and Ca traces and as a smaller peak in the S traces of all three samples. It had a molecular weight of 6.2 kDa as determined by SEC column calibration and confirmed using QTOF-MS to a mass of 6.574 kDa. Similar to the previous peak, it lies within the mass range of known plant MTs [33]. This is also the first significant peak present in the beetroot leaves sample. Between 22.1 and 25.3 min, a variety of peaks eluted in the Cd trace for beetroot leaves. The only two clearly distinguished peaks are the first and last peaks, with masses ranging from 6.2 to 3.7 kDa.

During this timeframe, using the QTOF-MS, 11 different types of PCs, in both the oxidized and reduced form, as well as with different terminal amino acids, were identified, as seen from Table 3 (more information on the compounds can be found in Table S8 in Online Resources 1). Initially, for the identification of PCs, the PyCDB database [37] was utilized to query the QTOF-MS measurements for the monoisotopic masses of the PCs. Then CMF-ID 4.0 [39] was used to perform in silico fragmentation of the analytes that were found to be present with a mass deviation of lower than 10 ppm and an area of at least 5000 a.u. A generally much higher presence of PCs in the beetroot leaf sample and a generally lower presence in the black-eyed bean and tigernut samples was observed. These findings also correlate with the ICP-MS/MS measurements. The PCs that were found included homophytochelatin, with alanine (Ala) as the terminal group; desglycine phytochelatin, lacking the terminal glycine; hydroxymethyl-phytochelatin, with serine (Ser); iso-phytochelatin with glutamate (Glu); and iso-phytochelatin with glutamine (Gln). Generally, shorter chain PCs were found in higher amounts, decreasing with longer chains. PCs with more than five repeating units were not found. The most abundant PC in all samples was the oxidized form of iso-PC2: (S-S)(γ-Glu-Cys)₂Gln. Generally, plant PCs are produced with a molecular weight range of 0.5–2.5 kDa and the observed PCs could therefore also be dimers or trimers.

**Table 3:** Identified PCs in the beetroot leaves sample using SEC-QTOF-MS in the retention time range of 22.1 to 25.3 min

RT/min	Name	m/z	Area/a.u.	Identification score by Schymanski [40]
18.83	(S-S)(gamma-Glu-Cys)_2_-Gly	538.1278	409,756	3
23.34	(S-S)(gamma-Glu-Cys)_3_-Gln	841.2167	1,936,625	3
23.36	(gamma-Glu-Cys)_3_-Gln	843.2323	531,543	3
23.41	(gamma-Glu-Cys)_3_-Ser	802.2058	88,668	3
23.41	(S-S)_2_(gamma-Glu-Cys)_4_-Ser	1030.226	21,350	3
23.43	(gamma-Glu-Cys)_3_-Ala	786.2108	24,812	3
23.43	(S-S)_2_(gamma-Glu-Cys)_4_-	943.1942	4661	3
23.44	(S-S)(gamma-Glu-Cys)_5_-Ala	1248.299	6752	3
23.48	(S-S)_2_(gamma-Glu-Cys)_4_-Ala	1014.231	11,679	3
24.13	(gamma-Glu-Cys)_2_-Glu	612.1645	5915	3
24.28	(gamma-Glu-Cys)_2_-	483.1219	116,378	3

The final peak observed in the Cd chromatogram (30.7 min) is only present in the beetroot leaves samples, and the Ca chromatogram of all three samples, eluting at 30.7 min. It has a molecular weight of 1.6 kDa as determined by the column calibration. In the timeframe of the peak (28–32 min) using the database of MassBank [38], seven different compounds were identified using QTOF-MS, as seen from Table 4, with a mass deviation of less than 10 ppm and an area of at least 5000. More information on the compounds can be found in Table S9 in Online Resources 1.

**Table 4:** Identified metabolites in the beetroot leaves sample using SEC-QTOF-MS in the retention time range of 28 to 32 min

RT/min	Name	Biochemical class	m/z	Area/a.u.	Identification score by Schymanski [40]
28.53	Tyrosine	Amino acids	182.0829	8,928,819	3
28.53	Coumaric acid	Phenolic acid	187.0002	4,944,417	3
28.70	Myricetin	Flavonoid	165.0546	565,015	3
29.00	Phospho-d-glyceric acid	Phosphorylated sugar alcohol	303.0499	6,380,548	3
29.07	Quercetin	Flavonoid	355.1022	760,705	3
31.35	Riboflavin	Vitamin	319.0448	14,534	3
31.37	Chlorogenic acid	Phenolic acid	377.1476	248,738	3

The analysis identified several compounds, all of which exhibited the potential to bind Cd^2^⁺ through various functional groups. These compounds were categorized into different classes based on their chemical structures and chelation capabilities. The aromatic amino acid tyrosine was identified, which can interact with metals through its amine and phenolic groups. Tyrosine has been shown to bind divalent metals like Cd, Ca, Sn, and Zn [41]. Further, the phenolic acids, coumaric acid and chlorogenic acid, were found. These compounds are strong chelators due to their hydroxyl and carboxyl groups, which enable effective metal binding. The flavonoids myricetin and quercetin were also present in the samples. These flavonoids contain multiple hydroxyl groups, which allow them to form stable complexes with metals. Quercetin [42] and myricetin [43] are recognized for their metal-binding properties, particularly with Ca, which is also visible from the Ca trace in Fig. 2 at 30.7 min. Finally, two additional compounds with chelation potential from the phosphorylated sugar alcohol and vitamin categories were identified: phospho-d-glyceric acid and riboflavin [44]. While direct evidence for Cd chelation of these compounds is limited, they are potential Cd chelators based on their known interactions with other bivalent metals.

**Fig. 4:**
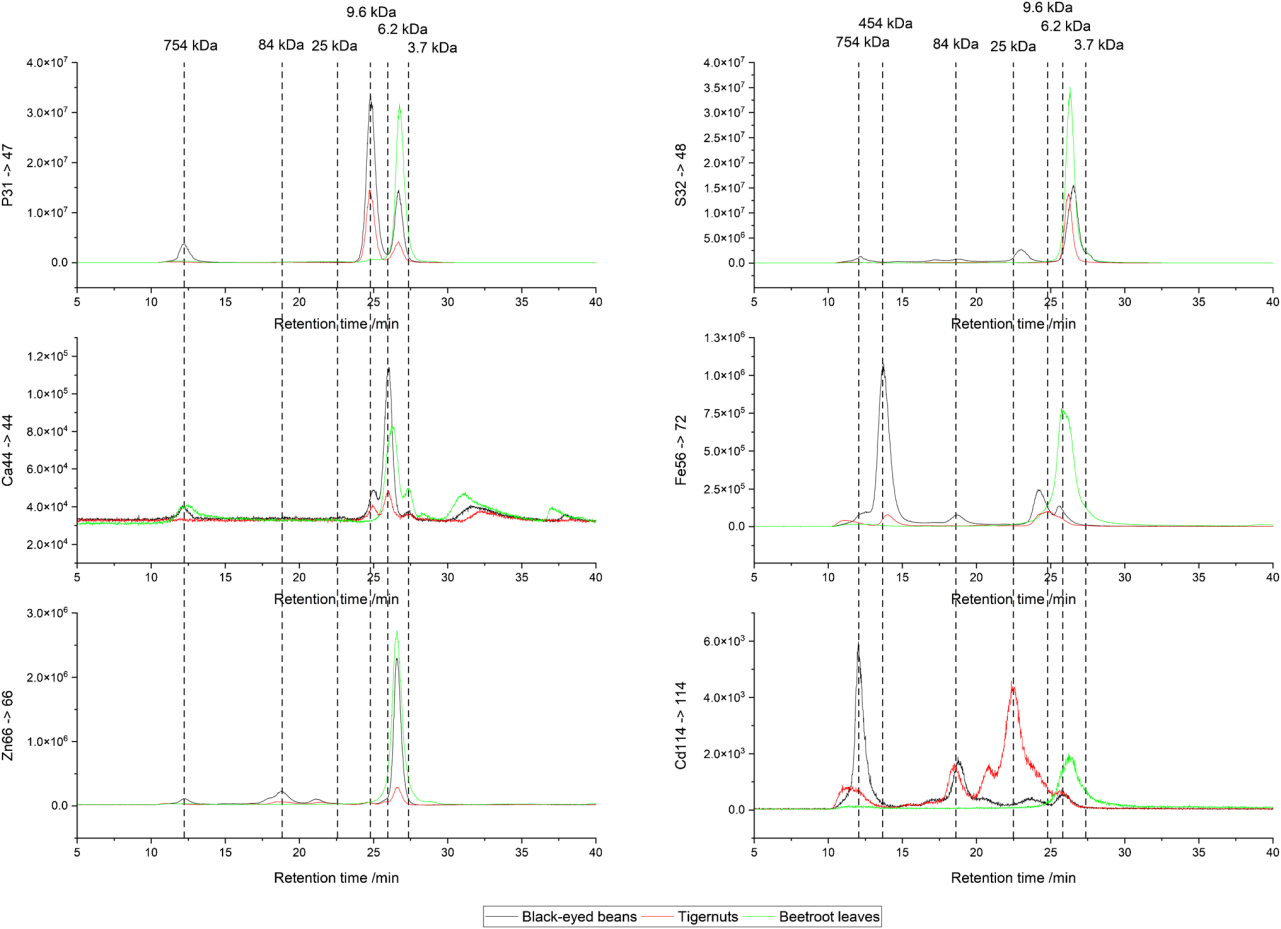
SEC separation and ICP-MS/MS detection of P, S, Ca, Fe, Zn, and Cd in cps (in one analytical run) using Superdex 200 Increase of the three samples black-eyed beans, tigernuts, and beetroot leaves. Vertical lines represent the peaks identified in the chromatograms for the Cd signal and the corresponding molecular weights (based on size calibration of the column)

With the Superdex 200 Increase column, larger proteins that were not separated using the Superdex 30 Increase column were separated, as seen from the SEC-ICP-MS/MS chromatograms in Fig. 4. The first Cd peak (11.9 min) with a calculated mass from the column calibration of 754 kDa is a feature in all element traces of the black-eyed bean, tigernut, and in small amounts in beetroot leaves samples. This peak, which represents the highest peak of Cd in the different samples, is particularly prominent in the black-eyed bean sample. Given its high mass, it is plausible to suggest that this peak is not representative of a single protein but rather a multi-protein complex. Furthermore, considering that it elutes above the optimal separation range of the column, it could also be a mix of multiple proteins that are too large, even for this column.

In the iron trace of black-eyed beans and tigernuts, a prominent peak is observed at 13.6 min. This compound, with a molecular mass of 454 kDa as determined by column calibration, is likely the ferritin complex due to its high molecular weight and specific iron-binding properties. In the literature, the molecular weight of phytoferritin is reported to range between 440 and 480 kDa, which aligns with the molecular weight observed [41]. Ferritin in plants plays a crucial role in iron storage and homeostasis, helping to regulate iron availability and protect cells from oxidative damage.

The second Cd peak (18.8 min) with a calculated mass from the column calibration of 84 kDa is present in the Cd, Fe, S, and Zn traces of the black-eyed beans and tigernut samples. Interestingly, the highest mass for Cd-chelating proteins reported in the literature is 60–70 kDa [17, 18], suggesting that this peak might represent a different protein or protein complex than previously reported. The third peak (22.5 min) had a calculated mass from the column calibration of 25 kDa. This peak, as well as the following peak, was also observed and discussed on the Superdex 30 Increase, indicating a consistent presence across different column calibrations.

To assess the relative importance of these HMW species, the total Cd area has been integrated. Compounds above 30 kDa accounted for approximately 73.0% of the total Cd area in black-eyed beans, 41.8% in tigernuts, and 11.7% in beetroot leaves. This area-based estimate shows that a substantial proportion of Cd can be associated with HMW compounds. This clearly supports the relevance of including the HMW range in the analysis and highlights the added value of the Superdex 200 column in capturing a more complete picture of Cd speciation in plant-based food matrices. Identification of these proteins could be achieved by a native measurement and consecutive fragmentation using QTOF-MS/MS. It is worth noting that the sensitivity of peak detection decreases for proteins with a mass exceeding 25 kDa due to decreased ionization efficiency, ion desolvation efficiency, as well as ion transmission [37]. To measure and identify compounds with a higher mass, a fractionation followed by a bottom-up enzymatic proteomics approach has to be used [38].

While the UV traces at 280 nm for the three samples, measured using both Superdex 30 Increase and Superdex 200 Increase, do not provide additional qualitative insights beyond those obtained by ICP-MS, they are presented in Fig. S5 in Online Resource 1, as UV detection remains essential for monitoring proteins larger than 40 kDa, which may not be efficiently detected by QTOF-MS due to its limited sensitivity for HMW species.

Overall, multielement speciation by SEC-ICP-MS/MS confirmed that Ca, Fe, and Zn are generally chelated to the same compounds as Cd, especially visible with the PCs in the range of 3.7–6.2 kDa range. Clear differences can be seen, though, as the essential trace elements prefer to bind to PCs with a higher molecular weight, at 6.2 kDa, while Cd has a higher binding affinity to lower molecular weight PCs, at 3.7 kDa. Persson et al. [19] also used SEC-ICP-MS and ESI-TOF-MS in barley roots to show that Cd-induced PC synthesis drives co-chelation not only of Cd but also of essential metals Zn, Cu, and Ni while Mn remained uncomplexed. They observed Zn-, Cu-, and Ni-species in SEC fractions of 0.7–1.8 kDa and 6.7–15 kDa, whereas only Cd species were detected in the range of 2.9–4.6 kDa, which is similar to our findings for Zn and Cd. This work extends the detectable size window up to 750 kDa and adds S and P traces to resolve phosphorylated proteins and phytic acid aggregates (9.6 kDa). Here, all elements bind to the phytic acid peak at 9.6 kDa, with Ca showing the highest intensity, then Zn, Fe, and lastly Cd. The 23.6 kDa MT fraction appears to bind only Cd.

## Conclusions

SEC-ICP-MS/MS in combination with SEC-QTOF-MS was demonstrated to be a suitable method for identifying Cd-chelating compounds. This is the first paper to compare three plant-based food samples (black-eyed beans, tigernuts, and beetroot leaves) for Cd speciation. Cd was found to bind to larger protein complexes, MTs, phytic acid, PCs, and (to lesser extend) to a variety of smaller molecules. QTOF-MS allowed the identification of phytic acid, 11 different PCs, and 7 smaller compounds. Cd speciation varied by matrix: black-eyed beans and tigernuts exhibited very similar binding profiles, whereas beetroot leaves displayed a markedly different distribution. This study presents significant advancements in Cd speciation analysis as it demonstrates that up to 73% of Cd binds to proteins larger than 30 kDa. This is also the first study, to our knowledge, to report Cd bound to HMW protein complexes up to 754 kDa. This highlights the need for an analytical approach as the one applied in this work, which combines two SEC columns.

The multielement speciation approach further showed that Ca, Fe, and Zn co-elute with Cd, apart from the MTs, revealing element-specific binding preferences. Furthermore, this is the first study to apply multielement speciation across such a broad biomolecular size range. These multi-element insights are not only analytically valuable but also biologically and nutritionally relevant, as they help elucidate the competitive interactions between Cd and essential nutrients and provide a clearer picture of how plants detoxify Cd through shared ligand pools. A systematic study of a larger number of plant-based foods with a larger variety of plant species and varieties is required, including also fresh fruits and vegetables.

Compared to other Cd speciation methods, the presented SEC-ICP-MS/MS-QTOF approach offers notable advantages and some limitations. Reversed-phase high-performance liquid chromatography (RP-HPLC) coupled with ICP-MS provides superior resolution for distinguishing closely related low molecular weight compounds but is less effective at detecting larger Cd-binding proteins [45]. Ion exchange chromatography (IEC) combined with MS achieves high specificity for Cd-ligand interactions by separating species based on charge differences [46]; however, it requires buffer systems that may interfere with MS detection. By incorporating SEC with ICP-MS/MS and QTOF-MS, the present methodology overcomes these limitations by expanding the detectable range of Cd species and providing both qualitative and potential quantitative insights into Cd interactions with proteins of varying sizes. Despite its advantages, the methodology has some inherent limitations. The resolution of SEC is limited when differentiating proteins of closely related molecular weights, which could impact the precise identification of specific Cd-binding species.

Another drawback of the method is the relatively long analysis time (2 × 60 min when using both SEC columns), which limits the number of samples that can be analyzed in 1 day. Future analytical work should further focus on the quantification of the Cd-containing species using SEC-ICP-MS/MS using either flow-injection [47] or online-isotope-dilution [48] techniques. The simultaneous detection of S, Cd, and other elements could in future allow the determination of the stoichiometry of metalloproteins and other compounds [49].

The developed method will significantly enhance the understanding of Cd speciation in plant-based foods. Additionally, it will help to identify and quantify the Cd species to which humans are exposed when performing simulated gastrointestinal digestion studies. This knowledge is crucial for toxicological studies, which, in combination with exposure assessments, will enable an improved estimation of the likelihood and severity of adverse health effects in humans [50–53].

## Supplementary Information

Below is the link to the electronic supplementary material.

Supplementary file1 (DOCX 1709 KB)

## Data Availability

The datasets generated during and analyzed during the current study are available from the corresponding author on reasonable request.
