# Supplementary material for: A novel approach for the identification of cadmium-chelating compounds in plant-based foods using SEC-ICP-MS/MS and SEC-QTOF-MS
Source: Anal Bioanal Chem. 2025 Jun 5;417(18):4147–61. doi: 10.1007/s00216-025-05931-y (PMC12276129; doi:10.1007/s00216-025-05931-y)
Supplement: Supplementary file 1 — Supplementary file1 (DOCX 1709 KB) [file 216_2025_5931_MOESM1_ESM.docx]

**Supplementary information**

**A Novel Approach for the Identification of Cadmium-Chelating Compounds in Plant-Based Foods Using SEC-ICP-MS/MS and SEC-QTOF-MS**

Julian Cardini^1[0000-0002-3157-7932]^, Jens J. Sloth^1[0000-0002-3636-8769]^

, Katrin Loeschner^1*[0000-0003-1741-8406]^

^1^ Research Group for Analytical Food Chemistry, National

Food Institute, Technical University of Denmark,

DK‑2800 Kgs. Lyngby, Denmark, * corresponding author: kals@food.dtu.dk

**Table S1:** Nutritional content of the studied hydrated samples per 100 g of sample (data from frida.fooddata.dk [1], USDA food data central [2] and Canadian Nutrient File (CNF) [3]).

| **Sample** | **Energy / kJ** | **Total Fat**  **/ g** | **of which saturated fat / g** | **Total Carbohydrate / g** | **Sugar / g** | **Protein / g** | **Fiber / g** |
| --- | --- | --- | --- | --- | --- | --- | --- |
| Black eye beans [3] | 490 | 0.71 | n.d. | 20.23 | n.d. | 8.13 | 3.6 |
| Beluga Lentils [2] | 610 | 0.63 | n.d. | 19 | n.d. | 11 | n.d. |
| Tigernut [2] | 502 | 7 | 2 | 14 | n.d. | 2 | 10 |
| Basmati Rice [3] | 544 | 0.19 | n.d. | 28.73 | 0.05 | 2.36 | n.d. |
| Sweet potato [1] | 302 | 0.3 | n.d. | 17.2 | 4.18 | 1.3 | 2.7 |
| Beetroot leaves [2] | 92 | 0,13 | n.d. | 4.33 | n.d. | 2 | 3.7 |

**Table S2:** Nutritional content of the studied dry samples per 100 g of sample (from the nutrition declaration on the package)

| **Sample** | **Energy / kJ** | **Total Fat**  **/ g** | **of which saturated fat / g** | **Total Carbohydrate / g** | **Sugar / g** | **Protein / g** | **Fiber / g** |
| --- | --- | --- | --- | --- | --- | --- | --- |
| Black eye beans | 1406 | 1.3 | 0.3 | 59.9 | 7.2 | 23.4 | 10.8 |
| Beluga Lentils | 1283 | 1.6 | 0.5 | 40.6 | 1.1 | 23.4 | 17 |
| Tigernuts | 1843 | 25 | 5.3 | 42 | 16 | 4 | 17 |
| Basmati Rice | 1490 | 0.7 | 0 | 80 | 4 | 9 | 4 |
| Sweet potato | 1439 | 0 | 0 | 78 | 18 | 6 | 3 |
| Beetroot leaves | 736 | 0.8 | 0 | 34.4 | 4 | 17.6 | 29.6 |

**Table S3:** Comparative analysis of full width at half maximum (FWHM) and resolution for different analytes (Horse heart myoglobin 16952 Da, Bovine insulin 5733 Da, Glufib 1570 Da, Raffinose 527 Da) under three different flow rates (0.1 ml/min, 0.075 ml/min, 0.05 ml/min) using the Superdex 30 Increase.

| **Analyte** | **0.1 ml/min** | | **0.075 ml/min** | | **0.05 ml/min** | |
| --- | --- | --- | --- | --- | --- | --- |
|  | **FWHM /min** | **Resolution /** | **FWHM /min** | **Resolution /** | **FWHM /min** | **Resolution /** |
| Horse heart myoglobin  16952 Da | 1.44 | 1.46 | 1.55 | 1.83 | 2.45 | 1.73 |
| Bovine insulin  5733 Da | 0.70 | 1.18 | 0.80 | 1.34 | 1.20 | 1.45 |
| Glufib  1570 Da | 0.85 | 1.91 | 1.00 | 1.83 | 1.40 | 2.05 |
| Raffinose  527 Da | 0.39 |  | 0.65 |  | 0.85 |  |

**Table S4:** Comparative analysis of full width at half maximum (FWHM) and resolution for different analytes (Thyroglobulin bovine 670 kDa, γ-globulins 150 kDa, Ovalbumin 44.3 kDa, Ribonuclease A type I-A 13.7 kDa and p-aminobenzoic acid 137 Da) under three different flow rates (0.1 ml/min, 0.075 ml/min, 0.05 ml/min) using the Superdex 200 Increase.

|  | **0.1 ml/min** | | **0.075 ml/min** | | **0.05 ml/min** | |
| --- | --- | --- | --- | --- | --- | --- |
| **Analyte** | **FWHM /min** | **Resolution /** | **FWHM /min** | **Resolution /** | **FWHM /min** | **Resolution /** |
| Thyroglobulin bovine | 1.3 | 1.68 | 1.40 | 2.04 | 2.21 | 1.97 |
| γ-globulins | 1.1 | 1.85 | 1.20 | 2.26 | 1.90 | 2.18 |
| Ovalbumin | 1.0 | 1.65 | 1.10 | 2.01 | 1.74 | 1.94 |
| Ribonuclease A type I-A | 0.6 | 12.45 | 0.60 | 15.18 | 0.95 | 14.64 |
| p-aminobenzoic acid | 1.5 |  | 1.6 |  | 2.53 |  |

**Table S5.** Intra-day repeatability presented as RSD (%) for retention time (RT for all detectors) and peak areas for UV (280 nm), QTOF-MS (total ion count), and ICP-MS/MS (isotopes detection across three different samples using Superdex 30 Increase: black-eyed beans, tigernuts, and beetroot leaves based on analysis of duplicates (N=2). The presented data includes the range and average of RSD for all detected peaks. The total number of detected peaks in each chromatogram is provided.

|  | **RT RSD / %** | | **UV peak areas RSD / %** | | **QTOF-MS peak areas RSD / %** | | **ICP-MS/MS peak areas RSD / %** | |
| --- | --- | --- | --- | --- | --- | --- | --- | --- |
| **Sample** | Range | Average | Range | Average | Range | Average | Range | Average |
| Black-eyed beans | 0-0.44 | 0.08 | 1.91-13.47 | 5.28 | 4.11-7.93 | 5.85 | 1.21-16.54 | 4.93 |
| No of peaks |  | | 17 | | 17 | | 28 | |
| Tigernuts | 0-0.77 | 0.10 | 0.08-6.15 | 2.52 | 0.04-6.09 | 3.01 | 0.07-16.45 | 2.89 |
| No. of peaks |  | | 15 | | 15 | | 22 | |
| Beetroot leaves | 0-0.93 | 0.10 | 0.32-14.77 | 5.05 | 1.06-12.68 | 5.05 | 0.01-13.66 | 2.38 |
| No. of peaks |  | | 10 | | 10 | | 29 | |

**Table S6.** Repeatability data for retention time (RT), UV signal and ICP-MS/MS detection methods across three different samples using Superdex 200 Increase: black-eyed beans, tigernuts, and beetroot leaves. The data includes the range and average of RSD values for each method and sample. No. of peaks represents the total number of peaks visible in the trace used for the calculations: UV signal at 280 nm and the total number of peaks detected for all element traces using ICP-MS/MS.

|  | **RT RSD / %** | | **UV signal RSD / %** | | **ICP-MS/MS RSD / %** | |
| --- | --- | --- | --- | --- | --- | --- |
| **Sample** | Range | Average | Range | Average | Range | Average |
| Black-eyed beans | 0-0.41 | 0.04 | 0.35-17.73 | 5.28 | 0.07-9.05 | 4.93 |
| No. of peaks |  |  | 17 | | 21 | |
| Tigernuts | 0.01-0.35 | 0.05 | 1.30-18.11 | 2.52 | 0.08-14.88 | 2.89 |
| No. of peaks |  |  | 15 | | 23 | |
| Beetroot leaves | 0.01-0.13 | 0.02 | 1.70-11.48 | 3.25 | 0.01-12.12 | 2.38 |
| No. of peaks |  |  | 10 | | 24 | |

**Table S7:** Intra-day and inter-day repeatability of element extraction efficiency expressed as relative standard deviation in %. This table presents the repeatability of element extraction efficiency for P, S, Ca, Fe, Zn, and Cd across three samples (black-eyed beans, tigernuts and beetroot leaves)

| **Intra-day 1** | **P / %** | **S / %** | **Ca / %** | **Fe / %** | **Zn / %** | **Cd / %** |
| --- | --- | --- | --- | --- | --- | --- |
| Black-eyed beans | 0.80 | 0.27 | 0.24 | 1.53 | 3.48 | 10.82 |
| Tigernuts | 2.29 | 1.37 | 3.41 | 0.86 | 1.61 | 4.42 |
| Beetroot leaves | 3.98 | 1.79 | 1.41 | 1.86 | 0.79 | 0.96 |
| **Average** | 2.36 | 1.14 | 1.69 | 1.42 | 1.96 | 5.40 |
| **Intra-day 2** |  |  |  |  |  |  |
| Black-eyed beans | 3.96 | 4.08 | 0.08 | 1.70 | 3.29 | 6.79 |
| Tigernuts | 2.59 | 0.70 | 3.44 | 2.70 | 0.81 | 2.24 |
| Beetroot leaves | 0.65 | 0.27 | 0.23 | 1.16 | 0.53 | 1.70 |
| **Average** | 2.40 | 1.68 | 1.25 | 1.85 | 1.54 | 3.58 |
| **Inter-day** |  |  |  |  |  |  |
| Black-eyed beans | 13.90 | 13.83 | 6.50 | 25.52 | 3.68 | 11.86 |
| Tigernuts | 11.00 | 5.78 | 6.46 | 3.90 | 6.45 | 2.69 |
| Beetroot leaves | 13.79 | 6.53 | 8.36 | 4.36 | 4.45 | 2.57 |
| **Average** | 12.90 | 8.72 | 7.11 | 11.26 | 4.86 | 5.71 |
|  |  |  |  |  |  |  |

**Table S8:** Different types of phytochelatins in the beetroot leaves samples identified using SEC-QTOF-MS

| **Name** | **[M+H]^+^** | **Deviation /ppm** | **RT /min** | **Area** | **Fragment 1** | **Deviation /ppm** | **Fragment 2** | **Deviation /ppm** | **Fragment 3** | **Deviation /ppm** |
| --- | --- | --- | --- | --- | --- | --- | --- | --- | --- | --- |
| (S-S)(gamma-Glu-Cys)2-Gly- | 538.1278 | 0.8 | 18.83 | 409756.1 | 492.1217 | 6.5 | 409.08462 | 2.6 | 407.06897 | 0.8 |
| (S-S)(gamma-Glu-Cys)3-Gln- | 841.2167 | 3.7 | 23.34 | 1936625 | 795.21062 | 2.2 | 609.16432 | 2.0 | n.d. | n.d. |
| (gamma-Glu-Cys)3-Gln- | 843.2323 | 2.7 | 23.36 | 531542.8 | 825.2212 | 1.9 | 797.2263 | 2.0 | 714.1891 | 5.2 |
| (gamma-Glu-Cys)3-Ser- | 802.2058 | 6.7 | 23.41 | 88667.77 | 784.1946 | 61.7 | 673.1626 | 4.3 | 441.1108 | 19.7 |
| (S-S)2(gamma-Glu-Cys)4-Ser- | 1030.226 | 9.2 | 23.41 | 21349.61 | 901.18309 | 5.2 | n.d. | n.d. | n.d. | n.d. |
| (gamma-Glu-Cys)3-Ala- | 786.2108 | 5.5 | 23.43 | 24811.66 | 768.1997 | 14.3 | 740.2048 | 11.5 | 437.1159 | 3.7 |
| (S-S)2(gamma-Glu-Cys)4- | 943.1942 | 7.3 | 23.43 | 4660.585 | 925.18309 | 3.0 | 897.1881 | 5.2 | 814.1511 | 3.9 |
| (S-S)(gamma-Glu-Cys)5-Ala- | 1248.299 | 9.8 | 23.44 | 6752.135 | 784.194 | 12.0 | n.d. | n.d. | n.d. | n.d. |
| (S-S)2(gamma-Glu-Cys)4-Ala- | 1014.231 | 7.2 | 23.48 | 11679.11 | 996.2202 | 4.4 | n.d. | n.d. | n.d. | n.d. |
| (gamma-Glu-Cys)2-Glu- | 612.1645 | 2.4 | 24.13 | 5915.08 | 566.15851 | 6.6 | 483.1214 | 0.2 | 465.1108 | 1.5 |
| (gamma-Glu-Cys)2- | 483.1219 | 1.8 | 24.28 | 116378.3 | 466.1002 | 1.5 | 465.1211 | 0.4 | 437.12039 | 0.2 |

**Table S9:** Different types of small molecules in the beetroot leaves sample identified using SEC-QTOF-MS

| **Name** | **Biochemical class** | **[M+H]+** | **Deviation /ppm** | **RT /min** | **Area** | **Fragment 1** | **Deviation /ppm** | **Fragment 2** | **Deviation /ppm** | **Fragment 3** | **Deviation /ppm** |
| --- | --- | --- | --- | --- | --- | --- | --- | --- | --- | --- | --- |
| Tyrosine | Amino acids | 182.0829 | 0.9 | 28.53 | 8928819 | 165.056 | 0 | 136.076 | 5.1 | n.d. | n.d. |
| Coumaric acid | Phenolic acid | 187.0002 | 4.8 | 28.53 | 4944417 | 147.0425 | 4.6 | 123.0423 | 1.3 | n.d. | n.d. |
| Myricetin | Flavonoid | 165.0546 | 1.2 | 28.7 | 565015 | 273.0394 | 3.1 | 153.0184 | 1.3 | n.d. | n.d. |
| Phospho-D-glyceric acid | Phosphorylated sugar alcohol | 303.0499 | 0.6 | 29 | 6380548 | 168.9343 | 3.4 | 145.93941 | 2.7 | n.d. | n.d. |
| Quercitin | Flavonoid | 355.1022 | 0.3 | 29.07 | 760705 | 274.0472 | 16.7 | 257.0444 | 0.3 | 153.0186 | 1.9 |
| Riboflavin | Vitamin | 319.0448 | 1.4 | 31.35 | 14534 | 243.0883 | 2.6 | 172.0877 | 1.5 | n.d. | n.d. |
| Chlorogenic acid | Phenolic acid | 377.1476 | 1.1 | 31.366 | 248738 | 303.0499 | 1.3 | 337.0860 | 4.4 | 163.037 | 2.4 |

**

**

**Fig. S1** Normalized S/N per ng/mL of P, S, Ca, Fe and Zn at varying oxygen flow rates ranging from 0.2 to 0.8 mL/min (matrix: 50 mM ammonium acetate)





**Fig. S2** Normalized S/N per ng/mL of Cd at varying oxygen flow rates ranging from 0.2 to 0.8 mL/min (matrix: 50 mM ammonium acetate)


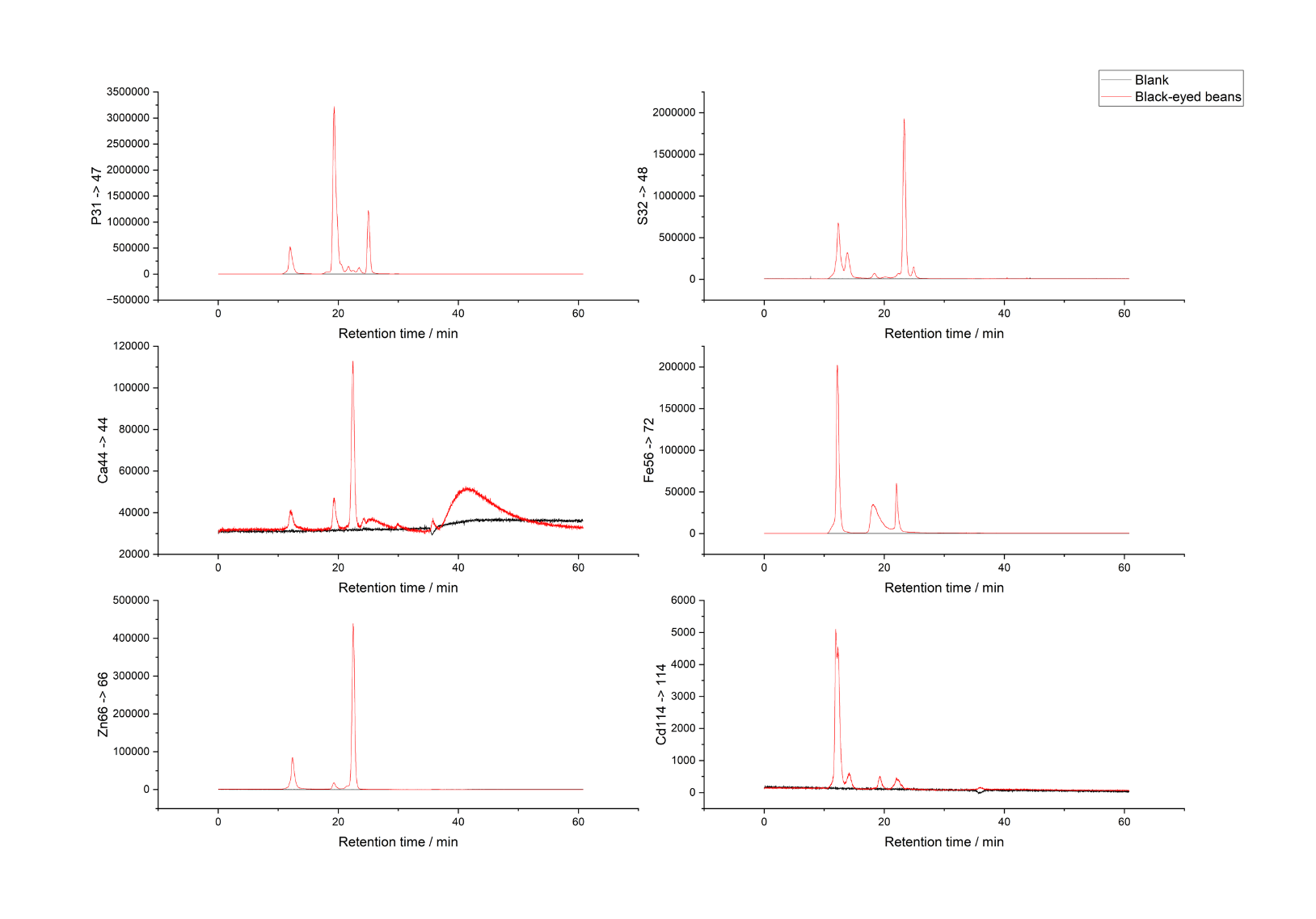


**Fig. S3** SEC separation and ICP-MS/MS detection of P, S, Ca, Fe, Zn and Cd (in one analytical run) using Superdex 30 - comparison of a UPW blank in comparison to the black-eyed beans sample. Notably, the lowest signal-to-noise ratio (S/N) was observed for Cd, where the S/N ranged from 3.1 for the smallest peak to 23.9 for the tallest peak. It is important to highlight that only an estimated mass of 0.3 ng of Cd was injected into the system (based on 31.7% recovery rate and Cd concentration of 102 µg/kg in the sample). This demonstrates that even extremely low amounts of Cd-chelates can be effectively separated and detected using this method.


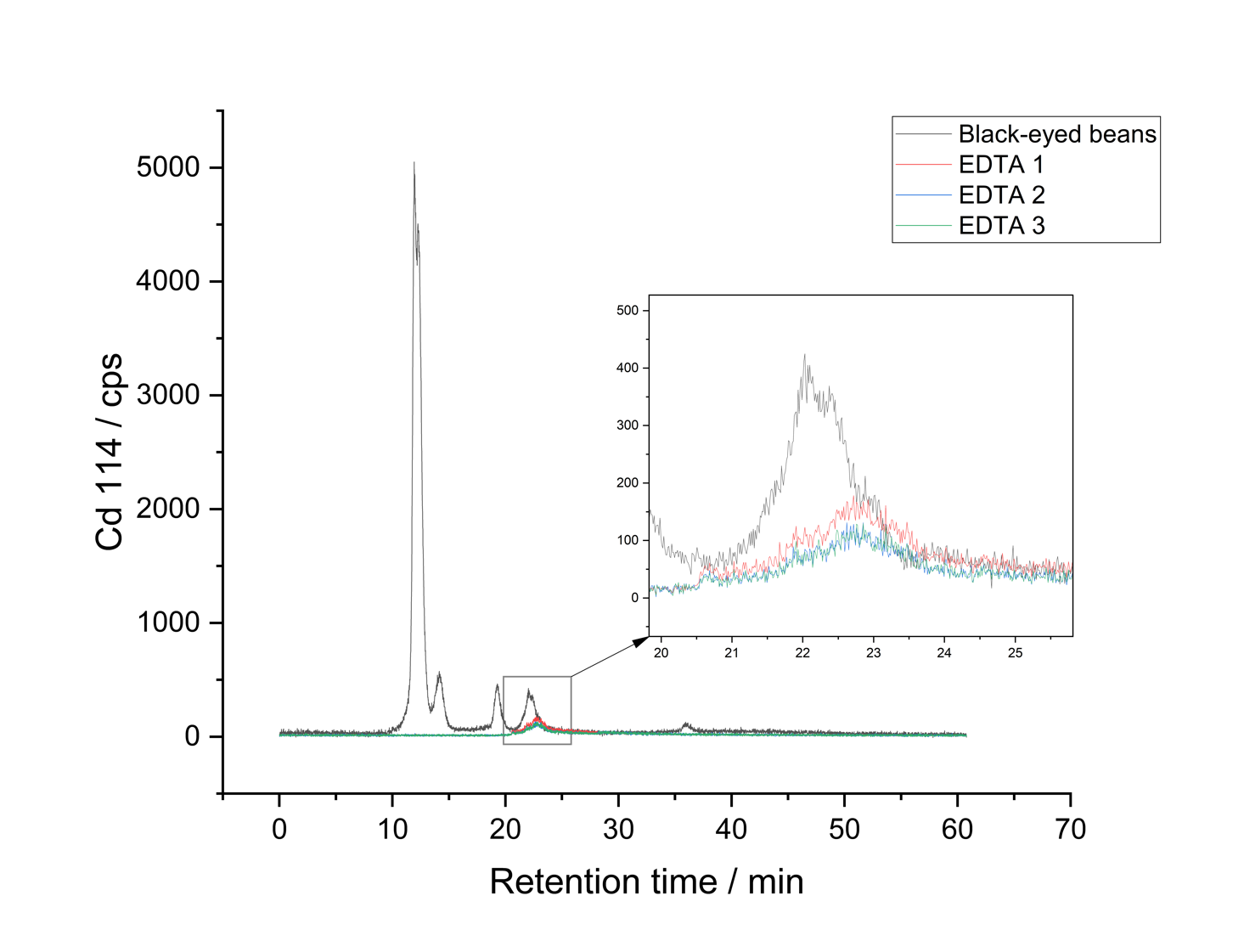


**Fig. S4** Comparison of Cd-trace of the black-eyed beans sample and three consecutive injections of 10 µL 5 mM EDTA in UPW using Superdex 30 increase column.





**Fig. S5** UV-traces at 280 nm of the black-eyed beans, tigernuts and beetroot leaves samples using Superdex 30 increase and Superdex 200 increase columns.
